# Dispersion from C^α^ or N^H^: 4D experiments for backbone resonance assignment of intrinsically disordered proteins

**DOI:** 10.1007/s10858-020-00299-w

**Published:** 2020-01-13

**Authors:** Helena Tossavainen, Santeri Salovaara, Maarit Hellman, Riikka Ihalin, Perttu Permi

**Affiliations:** 1 Department of Chemistry, Nanoscience Center, University of Jyväskylä, Jyväskylä, Finland; 2 Department of Biochemistry, University of Turku, Turku, Finland; 3 Department of Biological and Environmental Science, University of Jyväskylä, Jyväskylä, Finland

**Keywords:** *Aggregatibacter actinomycetemcomitans*, BilRI, Resonance assignment, Intrinsically disordered protein, IDP

## Abstract

Resonance assignment of intrinsically disordered proteins is remarkably challenging due to scant chemical shift dispersion arising from conformational heterogeneity. The challenge is even greater if repeating segments are present in the amino acid sequence. To forward unambiguous resonance assignment of intrinsically disordered proteins, we present iHACANCO, HACACON and (HACA)CONCAHA, three H^α^-detected 4D experiments with C^α^ as an additional dimension. In addition, we present (HACA)CON(CA)NH and (HACA)N(CA)CONH, new 4D H^α^-start, H^N^-detect experiments which have two N^H^ dimensions to enhance peak dispersion in a sequential walk through C′, N^H^ and H^N^, and provide more accurate N^H^/H^N^ chemical shifts than those that can be obtained from a crowded ^1^H, ^15^N-HSQC spectrum. Application of these 4D experiments is demonstrated using BilRI (165 aa), an outer-membrane intrinsically disordered protein from the opportunistic oral pathogen *Aggregatibacter actinomycetemcomitans*. BilRI amino acid sequence encompasses three very similar repeats with a 13-residue identical stretch in two of them.

**Electronic supplementary material:**

The online version of this article (10.1007/s10858-020-00299-w) contains supplementary material, which is available to authorized users.

## Introduction

*Aggregatibacter actinomycetemcomitans* is a Gram-negative opportunistic oral pathogen that is linked to periodontitis, infection of tissues supporting the teeth (for reviews, see Fine et al. 2006; Åberg et al. 2015; Fine et al. 2019). Although *A. actinomycetemcomitans* resides in subgingival multispecies biofilms, it is also able to migrate to underlying vessels and cause systemic diseases such as cardiovascular diseases (Kozarov et al. 2005; Hyvärinen et al. 2012). The host response to biofilms is mediated by inflammatory cytokines. In healthy junctional epithelium of tooth the balance of various cytokines and chemokines ensures that the host defence works appropriately. Some periodontal pathogens are able to impair the balance. *Porphyromonas gingivalis* can suppress the expression of chemokine interleukin(IL)-8 (Takeuchi et al. 2013) and *A. actinomycetemcomitans* biofilm is able to sequester and internalize IL-1β, IL-8 and IL-6, which leads to changes in biofilm composition and metabolic activity (Paino et al. 2011, 2012; Ahlstrand et al. 2017). It has been suggested that *A. actinomycetemcomitans* bacterial IL receptor I, BilRI is associated with this sequestering activity (Paino et al. 2013; Ahlstrand et al. 2017). BilRI is an outer membrane lipoprotein able to bind IL-1β, IL-8 and IL-10 and tumor necrosis factor (TNF)-α (Ahlstrand et al. 2017). Due to its rather low binding affinity it is assumed that BilRI acts by concentrating cytokines on cell membranes, which are then transferred to other components of the uptake system (Paino et al. 2013; Ahlstrand et al. 2017). We have engaged in the structural characterization of BilRI.

**Fig. 1:**
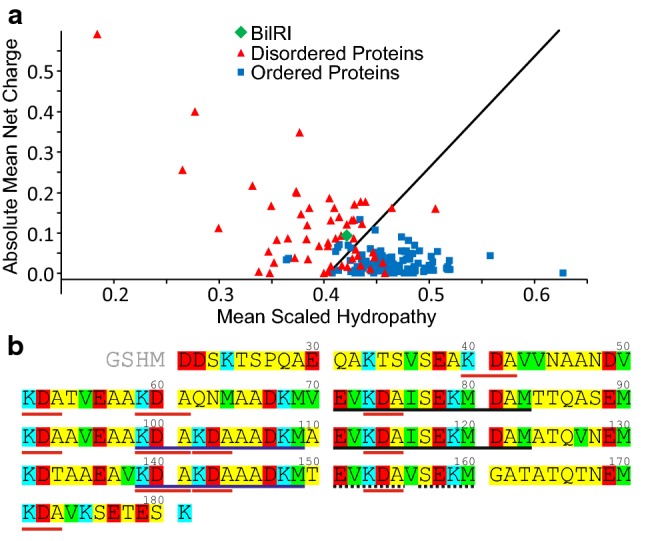
**a **Mean net charge-hydropathy plot of BilRI. Data for the plot were created with the PONDR predictor (https://www.pondr.com/). **b** Amino acid sequence of *A. actinomycetemcomitans* BilRI. Amino acids are classified by type: yellow, small hydrophilic (A, G, N, P, Q, S, T); green, hydrophobic (I, M, V); red, negatively charged (D, E) and blue, positively charged (K) amino acids. BilRI sequence does not contain C, F, H, L, R, W or Y. The longest 13-residue repetitive segments are underlined in black, and the broken underline indicates a very similar segment. The second longest 11-residue repetitive segments are anderlined in blue, and the triplet K-D-A, which recurs twelwe times in the sequence is underlined in red. The first four residues are a cloning artefact

BilRI is an intrinsically disordered protein (IDP), as demonstrated by its ^1^H, ^15^N HSQC spectrum, which displays very limited signal dispersion (Ahlstrand et al. 2017). In the H^N^ dimension their dispersion is only 0.63 ppm. This arises from a feature typical of IDPs, namely a biased amino acid composition with a pronounced number of polar or charged amino acids, low number of bulky hydrophobic amino acids and lack of aromatic amino acids (Dyson 2016). BilRI amino acid sequence is dominated by alanine (23%), lysine (14%) and aspartic acid (13%) residues. However, unlike the general trend (Dunker et al. 2001), BilRI sequence contains only one proline and one glycine. BilRI is positioned among IDPs in the mean net charge-hydropathy plot (Uversky et al. 2000) (Fig. 1a). The paucity in the variability of residue’s nearest neighbors narrows down the chemical shift range of a particular amino acid type, for example there are 12 Lys-Asp-Ala triplets in the sequence, which most likely results in close chemical shifts for the middle aspartic acid. Additionally, the BilRI sequence contains three very similar segments of about forty residues. The longest identical stretch is of 13 residues, present in two of these repeats, covering residues 71–82 and 111–122 (Fig. 1b).

The ^1^H, ^15^N HSQC spectra of IDPs typically being very challenging to scrutinize, the resonance assignment most often relies on spectra other than the classical H^N^-detected experiments widely used for folded proteins (for reviews see e.g. Sattler et al. 1999; Permi and Annila 2004). Indeed, the conventional approach based on the HNCACB and HN(CO)CACB/CBCA(CO)NH experiments that link the intra- and sequential ^13^C^α^ and ^13^C^β^ chemical shifts to ^1^H^N^ and ^15^N frequencies, are very inefficient for many IDPs due to severe clustering of aliphatic carbon chemical shifts for each residue type. Instead, correlation of ^15^N and ^13^C′ frequencies provide much better results for IDPs (Yao et al. 1997; Mäntylahti et al. 2009; Bermel et al. 2012) Another obstacle arises from the increasing chemical exchange rate of amide protons with water at alkali pH and/or elevated measurement temperature (Mäntylahti et al. 2010). Yet another challenge for the assignment originates from the abundancy of proline residues in IDPs. As an N-substituted residue, proline lacks the amide proton, which results in gaps during the resonance assignment procedure. Although this can be a benefit in the case of globular proteins, it significantly hampers the resonance assignment of disordered systems (Hellman et al. 2014). Several different approaches have been proposed to overcome these obstacles imposed during the assignment procedure. These include increase of dimensionality from conventional 3D to 4–7D spectra (Fiorito et al. 2006; Motáčková et al. 2010; Nováček et al. 2011; Kazimierczuk et al. 2013; Brutscher et al. 2015) as well as detection of non-exchangeable spins ^13^C′ and ^1^H^α^ instead of ^1^H^N^ (Bermel et al. 2006a, 2009; 2012; Mäntylahti et al. 2010, 2011; Permi and Hellman 2012). Our group has been resorting both to H^α^-start, H^N^-detect pulse schemes (Mäntylahti et al. 2009; Hellman et al. 2014) or complete H^α^-detection experiments (Mäntylahti et al. 2010, 2011; Permi and Hellman 2012) for the assignment of IDPs, which overcome hurdles associated with proline assignment and exchange broadening at alkali pH or elevated temperature. In addition to reduced susceptibility towards solvent exchange induced linebroadening, the H^α^ chemical shift is extremely valuable in structural analysis. The C^α^, H^α^ and C′ shifts are particularly sensitive to the ϕ/ψ angles of the protein backbone and thus the most informative in the estimation of secondary structure content in an IDP (Borcherds and Daughdrill 2018).

Comprehensive assignments allow for detailed, residue-specific analysis of structure and dynamics (Konrat 2014). There are therefore grounds for an extra effort towards a more comprehensive backbone resonance assignment. IDPs are often comprised of repetitive amino acid sequences and hence higher dimensionality in combination with high resolution offer superior results. However, the increased dispersion of signals should not be obtained, if possible, at expense of sensitivity. Here we present the resonance assignment of BilRI, whose demanding amino acid sequence necessitated development of a suite of 4D pulse sequences that offer superior signal dispersion with respect to their well-established 3D counterparts without indirect sampling associated sensitivity loss.

## Materials and methods

### Protein expression and purification

The gene encoding BilRI (residues 21–181) was cloned to pET15b vector (Novagen) into the NdeI and XhoI sites. This leads to soluble recombinant BilRI protein with N-terminal His-Tag with a thrombin cleavage site.

Production of ^13^C, ^15^N labeled BilRI was carried out by transforming plasmids into the BL21(DE3) cells. Cells were grown in M9 minimal media, supplemented with 1 g/l of ^15^NH_4_Cl and 2 g/l ^13^C-d-glucose as the sole nitrogen or nitrogen and carbon source, respectively. Cell culture was incubated at 37 °C and temperature was decreased to 16 °C when OD of the cell culture reached 0.4 and protein production was induced with 1 mM IPTG when OD of the cell culture reached 0.6. Cells were further incubated at 16 °C for 16 h and collected by centrifugation. Cells were disrupted with sonication and resulting supernatant was clarified by centrifugation with 30,000×*g*.

Clarified supernatant of BilRI was applied to the 1-mL His GraviTrap column (GE Healthcare) and the His-Tag was removed by thrombin protease (GE Healthcare) digestion according to the manufacturer’s instructions. Protease digested mixture was applied to His GraviTrap column. BilRI, without His-Tag, eluted with flow-through. Flow-through was concentrated to volume of 1 ml with Vivaspin 2 concentrator. Concentrated BilRI sample was applied into the Superdex 75 16/60 gel filtration column (GE Healthcare). Buffer used in gel filtration contained 20 mM sodium phosphate (pH 6.5) and 50 mM NaCl (NMR buffer). Fractions with pure BilRI were pooled and concentrated for NMR studies. The gel filtration was performed by using the ÄKTA Purifier FLPC purification system (GE Healthcare).

### NMR spectroscopy

BilRI NMR experiments were acquired using 0.5–1.0 mM ^15^N, ^13^C labeled protein samples in 5/95% D_2_O/H_2_O at pH 6.5. Chemical shifts were referenced to external 2,2,-dimethyl-2-silapentane-5-sulfonic acid (DSS). All data were acquired at 25 °C on a Bruker AVANCE III HD 800 MHz spectrometer, equipped with a TCI ^1^H/^13^C/^15^N cryoprobe. In addition to the new 4D experiments described here, the following experiments were used in the resonance assignment: 2D ^1^H, ^15^N-HSQC, constant time ^1^H, ^13^C-HSQC, ^13^C-detected 2D CON (Bermel et al. 2006b), 3D H^N^-detected HNCACB, CBCA(CO)NH, HNCO (reviewed in (Sattler et al. 1999; Permi and Annila 2004) and i(HACA)CO(CA)NH (Mäntylahti et al. 2009), 3D H^α^-detected HA(CA)CON, iHA(CA)NCO and (HACA)CON(CA)HA (Mäntylahti et al. 2010, 2011). All 3D/4D experiments were collected using non-uniform sampling (Table 1). Sampling densities were 25% for the 3D experiments and 7–20% for the 4D experiments. NMR data were processed with TopSpin 3.5 (Bruker Inc) and analyzed with CcpNmr Analysis v. 2.4.2 (Vranken et al. 2005). BilRI chemical shifts have been deposited to the BMRB database (www.bmrb.wisc.edu) with accession code 27824.

**Table 1:** Data acquisition parameters

Experiment	Points in F_1_ (ms)	Points in F_2_ (ms)	Points in F_3_ (ms)	Points in F_4_ (ms)	Sampling %	Number of scans
4D iHACANCO	80 (16.6) ^13^C′	84 (19.1) ^15^N	48 (3.9) ^13^C^α^	1024 (80.1) ^1^H^α^	10	4
4D HACACON	64 (13.2) ^15^N	64 (14.5) ^13^C′	48 (6.9) ^13^C^α^	1024 (80.1) ^1^H^α^	10	4
4D (HACA)CONCAHA	64 (13.2) ^13^C′	64 (14.5) ^15^N	48 (6.9) ^13^C^α^	1024 (80.1) ^1^H^α^	20	4
4D (HACA)CON(CA)NH	74 (26.3) ^13^C′	80 (24.7) ^15^N	82 (25.3) ^15^N	1024 (71.2) ^1^H^N^	7	4
4D (HACA)N(CA)CONH	74 (26.3) ^13^C′	80 (24.7) ^15^N	82 (25.3) ^15^N	1024 (71.2) ^1^H^N^	7	4
3D iHA(CA)NCO	116 (24.0) ^13^C′	200 (45.5) ^15^N	–	1024 (80.1) ^1^H^α^	25	8
3D HA(CA)CON	220 (50.0) ^15^N	230 (46.8) ^13^C′	–	1024 (80.1) ^1^H^α^	25	8
3D (HACA)CON(CA)HA	156 (32.3) ^13^C′	220 (50.0) ^15^N	–	1024 (80.1) ^1^H^α^	25	8

## Results and discussion

**Fig. 2:**
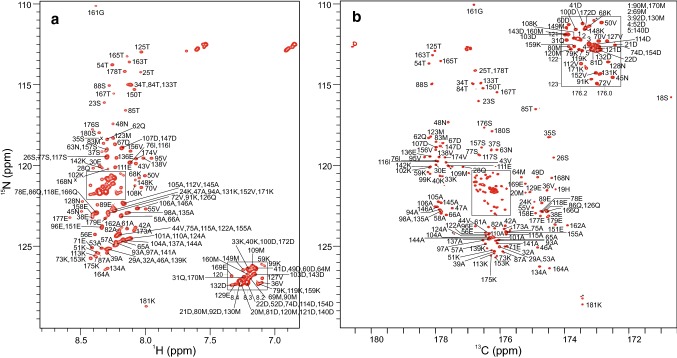
**a **2D ^1^H, ^15^N-HSQC spectrum of BilRI. The peaks are labeled with residue numbers and one-letter amino acid codes. Crosses indicate peaks found at lower contour levels. Residue numbering corresponds to that of whole BilRI protein (1–181) although the construct used was shorter (21–181). **b** 2D CON spectrum of BilRI. The peaks are labeled with residue number and amino acid code of the amide nitrogen in the C′-N^H^ pair. The peak of the only proline of the BilRI amino acid sequence, which resonates at 172.8 (^26^Ser C′), 138.1 (^27^Pro N^H^) ppm is not shown. Asterisks indicate impurities. Both 2D spectra were acquired at 800 MHz ^1^H frequency, 25 °C from a 1 mM BilRI sample at pH 6.5

Peaks in the BilRI 2D ^1^H, ^15^N-HSQC spectrum display overwhelming overlap (Fig. 2a) and a very narrow distribution in the H^N^ dimension. As it was later revealed, all but three of the 21 aspartic acid residues’ peaks are located in the middle region pile of peaks, 8.27–8.38, 121.2–121.9 ppm, together with 11 of the 13 methionine residues’ amide peaks. The Asp and Met α signals heavily overlap also in the ^1^H, ^13^C CT-HSQC (Suppl. Fig. S1). The CON spectrum, on the other hand, shows remarkably well dispersed signals (Fig. 2b) with peaks from all C′–N^H^ pairs present. We thus first attempted the assignment with spectra having the CON spectrum as the root spectrum, namely H^α^-detected experiments HA(CA)CON, iHA(CA)NCO and (HACA)CON(CA)HA (Mäntylahti et al. 2010, 2011). 3D H^N^-detected experiments HNCACB, CBCA(CO)NH, HNCO and i(HACA)CO(CA)NH (Mäntylahti et al. 2009) were acquired to collect H^N^, C′, C^α^ and C^β^ chemical shifts. With this set of seven 3D spectra, we assigned the majority of backbone resonances. However, there were several ambiguous assignments, in particular within the aforementioned repeating segments. Moreover, while precise H^α^ and C′ shifts were obtained from the 3D H^α^-detected experiments and the CON, due to heavy overlap, C^α^/C^β^ shifts were far more difficult to read from the HNCACB and CBCA(CO)NH spectra. Indeed, a precise C^α^/C^β^ chemical shift for 66% of the residues was obtained, while for H^α^, resolved peaks for 87% of the residues were observed (Suppl. Fig. S2).

To resolve these ambiguities and to extend the number of accurate chemical shifts, we resorted to 4D NMR spectroscopy. In order to bypass the C^α^ overlap problem in 3D H^N^-detected experiments and to establish direct connectivities between H^α^ and C^α^, and to provide dispersion to solve ambiguities arising from occasional overlap of C′, H^α^ resonances encountered in the 3D H^α^-detected experiments, we devised H^α^-detected 4D experiments with C^α^ as an additional dimension. In addition, we developed and employed new 4D H^α^-start, H^N^-detect experiments which can bridge stretches over single prolines similar to 3D experiments described in Hellman et al. (2014), but which have two N^H^ dimensions to enhance peak dispersion in a sequential walk through C′, N^H^ and H^N^, and to provide for more accurate N^H^/H^N^ chemical shifts than those that could be obtained from the crowded regions of the ^1^H, ^15^N HSQC.

### 4D iHACANCO, HACACON and (HACA)CONCAHA experiments

**Fig. 3:**
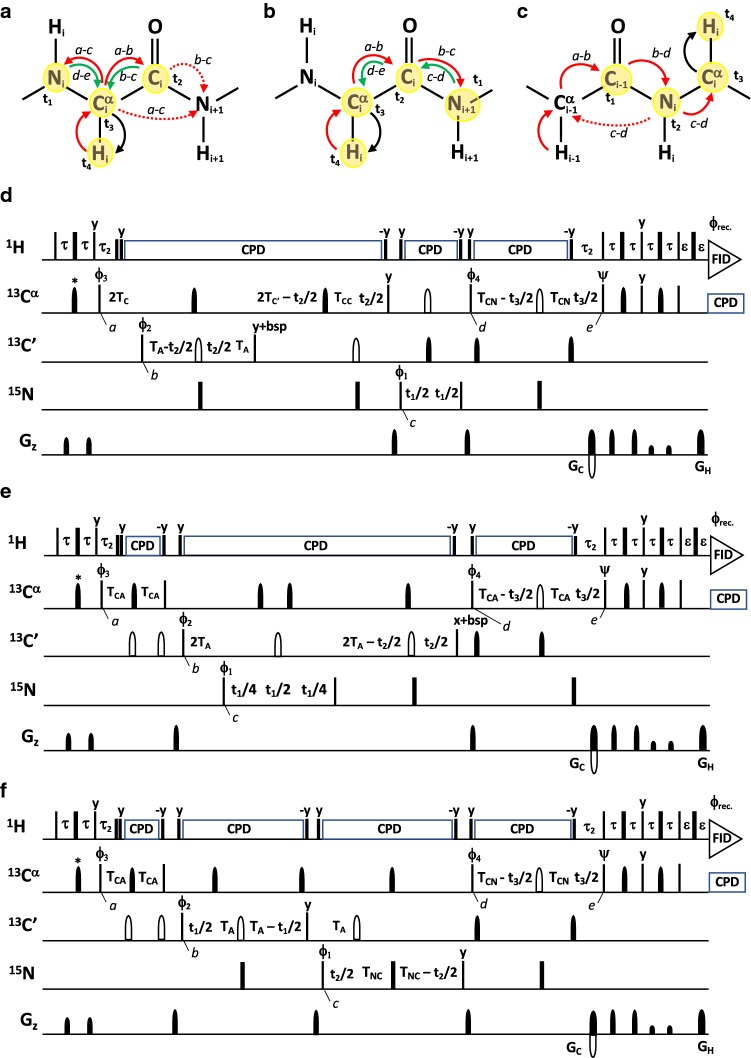
**a**–**c **Schematic presentation of magnetization transfer pathway during the 4D iHACANCO **d**, 4D HACACON **e** and 4D (HACA)CONCAHA **f** experiments. Red arrows indicate direct transfer pathway from ^1^H^α^(*i*) to ^15^N(*i*) or ^15^N(*i* + 1) whereas green arrows indicate a nested C^α^C′_Z_N_Z_ →N_Z_C^α^ transfer in d) known as the *intraresidual filter* (Permi 2002; Brutscher 2002) or highly selective C^α^C′_Z_N_Z_ →C^α^ transfer in e). Arrows indicate out-and-back type magnetization transfer, whereas one-way arrows represent coherence transfer route which is unidirectional. One-letter codes above the arrows indicate time points in the pulse sequence. **d** Intraresidual iHACANCO experiment to correlate ^1^H^α^(*i*), ^13^C^α^(*i*), ^13^C′(*i*) and ^15^N(*i*) chemical shifts, **e** the HACACON experiment, which correlates chemical shifts of ^1^H^α^(*i*), ^13^C^α^(*i*), ^13^C′(*i*) and ^15^N(*i* + 1) resonances. **f** The (HACA)CONCAHA experiment for correlating ^1^H^α^(*i*), ^13^C^α^(*i*), ^13^C′(*i*) and ^15^N(*i* + 1) resonances. Narrow and wide filled bars on ^1^H and ^15^N channels correspond to rectangular 90° and 180° pulses, respectively, applied with phase x unless otherwise stated. All ^13^C pulses are band-selective shaped pulses, denoted by filled narrow bars (90°) and filled and unfilled half ellipsoids (180°). Unfilled bars are applied on-resonance. The ^1^H, ^15^N, ^13^C′, and ^13^C^α^ carrier positions are 4.7 (water), 118 (center of ^15^N spectral region), 174 ppm (center of ^13^C′ spectral region), and 56 ppm (center of ^13^C^α^ spectral region). The ^13^C carrier is set initially to the middle of ^13^C′ region (174 ppm), shifted to ^13^C^α^ region (56 ppm) prior to 90° ^15^N pulse ϕ_1_ in scheme d). In scheme e) and f), the carrier is initially at 56 ppm and shifted to 174 ppm prior to 90° ^13^C pulse ϕ_2_, and shifted back to 56 ppm before 90° ^13^C pulse ϕ_4_. The first band-selective 180° ^13^C pulse, refocusing ^13^C^α^ magnetization (56 ppm, denoted with an asterisk) had duration of 788 μs at 800 MHz. Other band-selective 90° and 180° pulses for ^13^C^α^ (56 ppm) and ^13^C′ (174 ppm) were applied with durations of 240.0 μs and 192.0 μs at 800 MHz, respectively. Band-selective 90° and 180° pulses for ^13^C′/^13^C^α^ have the shape of Q5 and Q3 (Emsley and Bodenhausen 1992) and duration of 240.0 μs and 192.0 μs at 800 MHz, respectively. The adiabatic 180° Chirp broadband inversion pulse for inverting ^13^C^α^ and ^13^C′ magnetization in the middle of t_1_ period had duration of 500 μs at 800 MHz (Böhlen and Bodenhausen 1993). The Waltz-65 sequence (Zhou et al. 2007) with strength of 4.17 kHz was employed to decouple ^1^H spins. The GARP (Shaka et al. 1985, 1987) with field strength of 4.55 kHz was used to decouple ^13^C during acquisition. Delay durations: τ = 1/(4*J*_HC_) ~ 1.7 ms; τ_2_ = 3.4 ms (optimized for non-glycine residues) or 2.2–2.6 ms (for observing both glycine and non-glycine residues); ε = duration of G_H_ + field recovery ~ 0.4 ms; 2T_C_ = 1/(2*J*_C_α_C′_) ~ 9.5 ms; T_CA_ = 1/(6*J*_C_α_C′_) ~ 3.3 ms; T_A_ = 1/(4*J*_C′N_) ~ 16.6 ms; T_C′_ = T_C_ + T_CC_; T_CC_ = 1/(*J*_C_α_C_β)–1/(4*J*_C′N_)–1/(2*J*_C_α_C′_) ~ 0–2.5 ms; T_NC_ ~ 14 ms; T_CN_ ~ 14 ms; T_N_ ~ 14 ms. Maximum *t*_1_, *t*_2_ and *t*_3_ are restrained in scheme d, *t*_2,max_ < 2.0*T_C′_, *t*_3,max_ < 2.0*T_CN_, in scheme e, *t*_2,max_ < 4.0*T_A_, *t*_3,max_ < 2.0*T_CA_, in scheme f, *t*_1,max_ < 2.0*T_A_, *t*_2,max_ < 2.0*T_NC_, *t*_3,max_ < 2.0*T_CN_. Frequency discrimination in ^15^ N and ^13^C′ dimensions is obtained using the States-TPPI protocol (Marion et al. 1989) applied to ϕ_1_ and ϕ_2_, respectively, whereas the quadrature detection in ^13^C^α^ dimension is obtained using the sensitivity-enhanced gradient selection (Kay et al. 1992; Schleucher et al. 1994). The echo and antiecho signals in ^13^C^α^ dimension are collected separately by inverting the sign of the G_C_ gradient pulse together with the inversion of ψ, respectively. Phase cycling: ϕ_1_ = x, − x; ϕ_2_ = 2(x), 2(− x); ϕ_3_ = 4(x), 4(− x); ϕ_4_ = x; ψ = x; rec. = x, 2(− x), x, − x, 2(x), − x. Selective 180° pulse for ^13^C^α^ in the middle of delay 2T_A_ induces a Bloch-Siegert shift to ^13^C′ magnetization, a careful adjustment of phase (bsp) of the last ^13^C′ 90° (phase y) pulse is necessary in scheme d). Gradient strengths and durations: G_C_ = 13 k G/cm (1.6 ms), G_H_ = 13 k G/cm (0.4 ms). The pulse sequences code and parameter file for Bruker Avance system are available from authors upon request

The proposed 4D iHACANCO, HACACON and (HACA)CONCAHA experiments are extensions of their established 3D counterparts (Mäntylahti et al. 2010, 2011) with additional sampling on the fourth ^13^CA dimension (Fig. 3). The coherences flow through the 4D iHACANCO, HACACON and (HACA)CONCAHA experiments in Eqs. 1, 2, 3:

1 $${}^{1}H^{\alpha } (i)\xrightarrow{{2\tau \left( {^{1} J_{{H\alpha C\alpha }} } \right)}}{}^{{13}}C^{\alpha } (i)\xrightarrow{{2T_{C} \left( {{}^{1}J_{{C\alpha N}} ,{}^{2}J_{{C\alpha N}} ,^{1} J_{{C\alpha C'}} } \right)}}{}^{{13}}C^{\prime}(i)\left[ {2T_{A} - t_{2} ;{}^{1}J_{{C^{\prime}N}} ,{}^{1}J_{{C\alpha N}} ,{}^{2}J_{{C\alpha N}} } \right] \to {}^{{13}}C^{\alpha } (i)\left[ {2T_{{C^{\prime}}} - t_{2} ;{}^{1}J_{{C^\alpha N}} ,{}^{2}J_{{C\alpha N}} ,{}^{1}J_{{C\alpha C^{\prime}}} } \right] \to {}^{{15}}N(i)[t_{1} ] \to {}^{{13}}C^{\alpha } (i)\left[ {2T_{CN} - t_{3} ;{}^{1}J_{{C^\alpha N}} ,{}^{2}J_{{C\alpha N}} } \right]\xrightarrow{{4\tau \left( {^{1} J_{{H\alpha C\alpha }} } \right)}}{}^{1}H^{\alpha } (i)[t_{4} ]$$

2 $${}^{1}{\text{H}}^{\alpha } (i)\xrightarrow{{2\tau \left( {^{1} J_{{H\alpha C\alpha }} } \right)}}{}^{{13}}C^{\alpha } (i)\xrightarrow{{2T_{CA} \left( {^{1} J_{{C\alpha C'}} } \right)}}{}^{{13}}C^{\prime}(i)\xrightarrow{{2T_{{\text{A}}} \left( {^{1} J_{{{\text{C}}'{\text{N}}}} } \right)}}{}^{{15}}N(i + 1)[t_{1} ] \to {}^{{13}}C^{\prime}(i)\left[ {2T_{A} - t_{2} ;{}^{1}J_{{C^{\prime}N}} } \right] \to {}^{{13}}C^{\alpha } (i)\left[ {2T_{{CA}} - t_{3} ;{}^{1}J_{{C\alpha C^{\prime}}} } \right]\xrightarrow{{4\tau \left( {^{1} J_{{H\alpha C\alpha }} } \right)}}{}^{1}H^{\alpha } (i)[t_{4} ]$$

3 $${}^{1}{\text{H}}^{\alpha } (i - 1)\xrightarrow{{2\tau \left( {^{1} J_{{H\alpha C\alpha }} } \right)}}{}^{{13}}C^{\alpha } (i - 1)\xrightarrow{{2T_{CA} \left( {^{1} J_{{C\alpha C'}} } \right)}}{}^{{13}}C^{\prime}(i - 1)\left[ {2T_{A} - t_{1} ;{}^{1}J_{{C^{\prime}N}} } \right] \to {}^{{15}}N(i)\left[ {2T_{{NC}} - t_{2} ;{}^{1}J_{{C\alpha N}} ,{}^{2}J_{{C\alpha N}} ,{}^{1}J_{{C^{\prime}N}} } \right] \to {}^{{13}}C^{\alpha } (i)\left[ {2T_{{CN}} - t_{3} ;{}^{1}J_{{C\alpha N}} ,{}^{2}J_{{C\alpha N}} } \right]\xrightarrow{{4\tau \left( {^{1} J_{{H\alpha C\alpha }} } \right)}}{}^{1}H^{\alpha } (i)[t_{4} ]$$

respectively. All experiments start with the ^1^H^α^(*i*)→ ^13^C^α^(*i*) transfer, and the density operator immediately after the ϕ_3_ pulse is described as H^α^_z_(*i*)C^α^_z_(*i*) (time point *a*). Subsequently, the magnetization is transferred to the ^13^C′ spin followed by the labeling of ^13^C′ chemical shift in t_1_ or t_2_. The relevant density operator after the ϕ_2_ pulse in all experiments is described as C^α^_z_(*i*)C′_y_(*i*) (time point *b*). Next, the desired coherence is transferred to the ^15^N spin of the sequential residue in HACACON and (HACA)CONCAHA experiments, described with the density operator C^α^_z_(*i*)C′_z_(*i*)N_y_(*i* + 1) (time point *c*). In the iHACANCO experiment, the magnetization is solely transferred to the ^15^N spin within the residue, described with the density operator C^α^_z_(*i*)C′_z_(*i*)N_y_(*i*) (time point *c*). After labeling the ^15^N chemical shifts in t_1_ (or t_2_), the magnetization is transferred to the ^13^C^α^ coherence after the ϕ_4_ pulse. The relevant density operators (time point *d*) are C^α^_y_(*i*)N_z_(*i*) for iHACANCO, C^α^_y_(*i*)C′_z_(*i*) for HACACON and C^α^_y_(*i*)N_z_(*i*) for (HACA)CONCAHA schemes. The ^13^C^α^ chemical shift is labeled during the t_3_ period between time points *d*–*e*.

While the HACACON (Fig. 3b) is the conventional out-and-back experiment, the (HACA)CONCAHA and iHACANCO utilize the intraresidual filter for the selective ^13^C^α^(*i*)→ ^15^N(*i*) transfer (Permi 2002; Mäntylahti et al. 2010, 2011). Especially in the iHACANCO experiment, the magnetization transfer is nested and further clarification is delivered in the following. After converting magnetization to the H^α^_z_(*i*)C^α^_z_(*i*) coherence (time point *a*), the ^1^J_CαC′_, ^1^J_CαN_, ^2^J_CαN_ and ^1^J_CαCβ_ couplings are active during the time interval (2T_C_ + 2T_A_ + 2T_C′_ + T_CC_) = 52–57 ms, which converts it to the C^α^_z_(*i*)N_y_(*i*) coherence (time point *c*). However, during the delay 2T_CC_, that can be selected to be 0–5 ms based on the relaxation properties of ^13^C^α^ spins, only ^1^J_CαCβ_ is active. This is to maximize the transfer efficiency during the (2T_C_ + 2T_A_ + 2T_C′_ + T_CC_) delay. Of note, to avoid chemical shift evolution of ^13^C^α^–^13^C′ multiple-quantum coherence during 2T_A_ –t_2_, an additional but opposite frequency labeling period for ^13^C^α^ has been implemented in the 2T_C′_ period. Efficiently, only the chemical shift evolution of ^13^C′ will take place during t_2_, but the attainable resolution is limited by 2T_C′_ (= 2T_C_ + T_CC_) i.e. t_2,max_ is 19–22 ms, depending on the setting of 2T_CC_ (0–5 ms).

Thus, the 4D iHACANCO, HACACON and (HACA)CONCAHA experiments yield correlations at ω_HA(i)_, ω_CA(i)_, ω_C′(i)_, ω_N(i)_; ω_HA(i)_, ω_CA(i)_, ω_C′(i)_, ω_N(i+1)_, and ω_HA(i)_, ω_CA(i)_, ω_C′(i–1)_, ω_N(i)_ frequencies, respectively. Given that frequency labeling of ^13^C^α^ chemical shifts is implemented in a constant-time manner, without lengthening the actual pulse sequence and incorporating sensitivity enhanced gradient echo in t_3_, there is no sensitivity loss involved in increasing the dimensionality of these H^α^-detected experiments. Hence, the coherence transfer efficiencies provided with the corresponding 3D experiments by Mäntylahti et al. (2010, 2011) are directly comparable to 4D implementations shown in Fig. 3. Indeed, by taking into account typical values of one-bond couplings ^1^*J*_CαC′_ = 53 Hz, ^1^J_C′N_ = 15 Hz, and ^1^*J*_CαCβ_ = 35 Hz, and the average random coil values for one-bond (^1^*J*_CαN_ = 10.6 Hz) and two-bond (^2^*J*_CαN_ = 7.5 Hz) couplings between backbone ^13^C^α^ and ^15^N spins (Delaglio et al. 1991), as well as transverse relaxation times (T_2_) for ^13^C^α^ (= 100 ms), ^13^C′ (= 200 ms) and ^15^N (= 200 ms) spins, we can estimate coherence transfer efficiencies for these experiments in IDPs (Mäntylahti et al. 2010, 2011). The HACACON is superior in sensitivity (I ~ 0.28) in comparison to iHACANCO and (HACA)CONCAHA experiments, with coherence transfer efficiencies of 0.22 and 0.18, respectively. Particularly, for the assignment of prolines, sensitivities of iHACANCO and HACACON are superior to the (HACA)CONCAHA scheme, which yields coherence transfer of 0.026 for proline residues. The sensitivity loss is associated with the ^15^N(*i*)→ ^13^C^α^(*i*) transfer, 2T_NC_, during which the ^15^N magnetization is further modulated by ^1^J_NCδ_ coupling interaction in prolines.

**Fig. 4:**
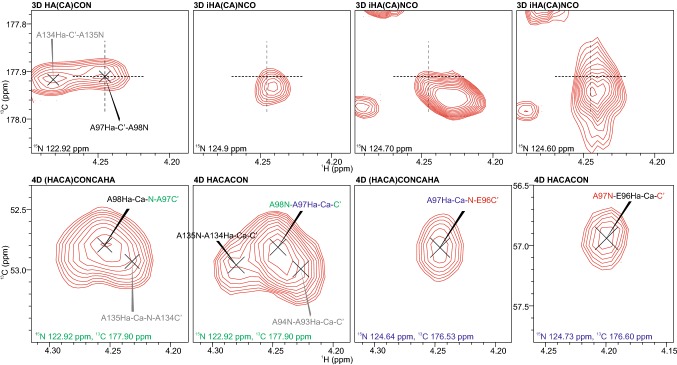
Problematics in resonance assignment with 3D H^α^-detected experiments can be resolved with 4D HACACON and 4D (HACA)CONCAHA experiments. The upper row of 2D planes from 3D HA(CA)CON and iHA(CA)NCO spectra shows that the sequential walk from ^97^Ala to ^96^Glu is ambiguous because the ^97^Ala Ha, C′ shift pair observed in the HA(CA)CON can be found in several planes of the iHA(CA)NCO and is overlapping with other peaks. While there is overlap in the 4D spectra also, the planes are far more easily interpreted and allow for unambiguous assignment of ^98^Ala-^97^Ala-^96^Glu. Grey labels mark peaks with maximum in adjacent ^15^N or ^13^C planes

Figure 4 compares 3D H^α^-detected with the new 4D spectra: problems associated with multifold overlap and ambiguities in choosing the right sequential connection when using 3D spectra can be surpassed, and the assignment procedure expedited by extending frequency labeling to C^α^.

With the help of these H^α^-detected 4D experiments, 76% of the α correlations of were successfully assigned (Suppl. Fig S2). Considering that some N, C′ resonances are separated by less than 0.05 ppm in the CON spectrum, e.g. corresponding resonances within^73/113^KDAISE^78/118^ differ only by 0.03–0.04 ppm, all peaks were not expected to be resolved in spectra with spectral resolutions of 0.11 (^15^N) and 0.05 (^13^C) ppm. The H^α^, C^α^ shifts were as ineffective in providing the needed dispersion, the smallest peak separations being comparable, e.g. the ^76/116^Ile alpha peaks differ by only 0.01 and 0.05 ppm in ^1^H and ^13^C, respectively, and those of ^77/117^Ser even less (Suppl. Fig. S1).

### 4D (HACA)CON(CA)NH and (HACA)N(CA)CONH experiments

**Fig. 5:**
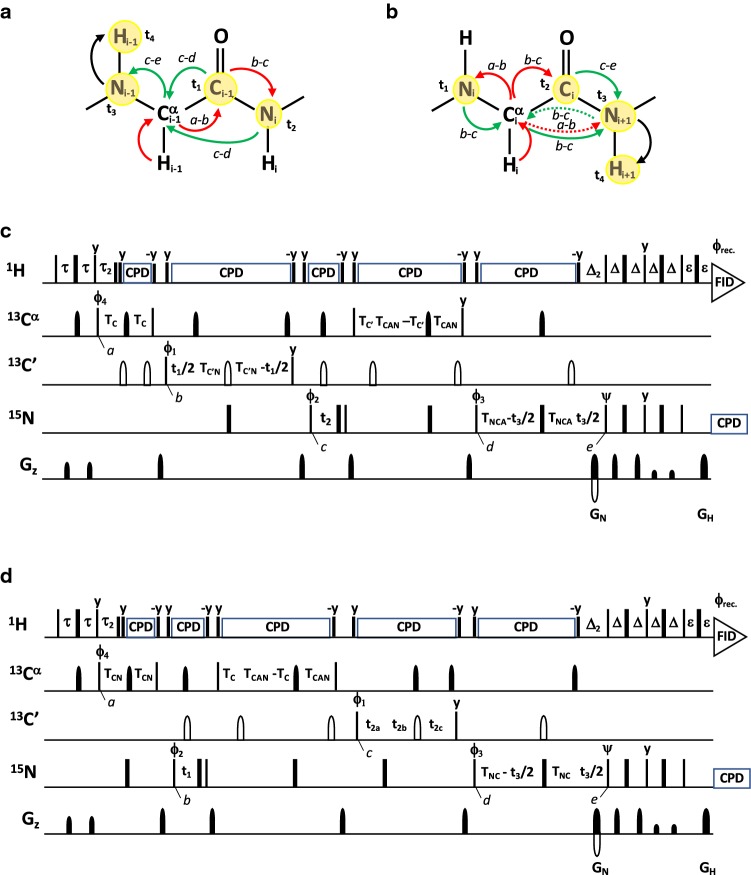
**a** and **b** schematic representation of the magnetization transfer pathways during the **c** 4D (HACA)CON(CA)NH and **d** 4D (HACA)N(CA)CONH experiments, respectively. Red arrows highlight direct transfer pathway from ^1^H^α^(*i*) to ^15^N(*i*) or ^15^N(*i* + 1) whereas green arrows indicate a nested C^α^C′_Z_N_Z_ →N_Z_C^α^ transfer in **c** or N_Z_C^α^ →C^α^C′N_Z_ transfer in **d**, known as the *intraresidual filter* (Brutscher 2002; Permi 2002). Black arrows stand for the coherence order selective coherence transfer, COS-CT (Kay et al. 1992; Schleucher et al. 1994). Arrows with solid line denote the actual magnetization transfer pathway, whereas an arrow with a broken line displays coherence transfer that is suppressed by the *intraresidual filter*. The 4D (HACA)CON(CA)NH experiment establishes connectivities between ^1^H^N^(*i*–1) (t_4_), ^15^N(*i*–1) (t_3_), ^15^N(*i*) (t_2_), and ^13^C′(*i*–1) (t_1_) frequencies. The 4D (HACA)N(CA)CONH scheme correlates ^1^H^N^(*i* + 1) (t_4_), ^15^N(*i* + 1) (t_3_), ^13^C′(*i*) (t_2_) and ^15^N(*i*) (t_1_) frequencies. These two experiments allow also bridging the gap between XPX motifs, where X is any other residue except proline. Narrow and wide bars correspond to 90° and 180° pulses for ^1^H and ^15^N, respectively. Pulses are applied with phase x unless otherwise marked. All ^13^C pulses are band-selective shaped pulses, denoted by filled narrow bars (90°) and filled and unfilled half ellipsoids (180°). Unfilled half ellipsoids denote on-resonance 180° pulses. The ^1^H, ^15^N, ^13^C′, and ^13^C^α^ carrier positions are 4.7 (water), 118 (center of ^15^N spectral region), 174 ppm (center of ^13^C′ spectral region), and 56 ppm (center of ^13^C^α^ spectral region). The ^13^C carrier resides at 174 ppm throughout the experiments. Band-selective 90° and 180° pulses for ^13^C′/^13^C^α^ have the shape of Q5 and Q3 (Emsley and Bodenhausen 1992) and duration of 240.0 μs and 192.0 μs at 800 MHz, respectively. The Waltz-65 sequence (Zhou et al. 2007) with strength of 4.17 kHz was employed to decouple ^1^H spins. The GARP-4 (Shaka et al. 1985) with field strength of 1.14 kHz was used to decouple ^15^N during acquisition. Delay durations: τ = 1/(4*J*_HC_) ~ 1.7 ms; τ_2_ = 2.6 ms (for the scheme c) or 2.2 ms (for the scheme d); ∆ = 1/(4*J*_HN_) ~ 2.7 ms; ∆_2_ = 1/(2*J*_HN_) ~ 5.5 ms; ε = duration of G_H_ + field recovery ~ 1.2 ms; 2T_C_ = 1/(6*J*_C_α_C′_) ~ 6.2 ms or 28 ms; 2T_C′_ ~ 9.4 ms; 2T_C′N_ ~ 33 ms; 2T_NCA_ ~ 29 ms; The ^13^C^α^^15^N transfer delay 2T_CAN_ ~ 50–56 ms to suppress the auto-correlated pathway, or 2T_CAN_ ~ 25 ms for observing both sequential and auto-correlated cross-peaks. Maximum *t*_1_ and *t*_3_ is restrained in scheme c, *t*_1,max_ < 2.0*T_C′N_, *t*_3,max_ < 2.0*T_NCA_, and *t*_3_ is restricted in scheme d, *t*_3,max_ < 2.0*T_NC_. Frequency discrimination in ^13^C′ and ^15^N dimensions is obtained using the States-TPPI protocol (Marion et al. 1989) applied to ϕ_1_ and ϕ_2_. Frequency discrimination in the second, t_3_, ^15^N dimension is accomplished by the COS-CT implementation (Kay et al. 1992; Schleucher et al. 1994). The echo and antiecho signals in the ^15^N dimension are collected separately by inverting the sign of the G_N_ gradient pulse together with the inversion of ψ, respectively. Phase cycling: ϕ_1_ = x, − x; ϕ_2_ = 2(x), 2(− x); ϕ_3_ = x; ϕ_4_ = 4(x), 4(− x); ψ = x; ϕ_rec._ = x, 2(− x), x, − x, 2(x), − x. Gradient strengths and durations: G_N_ = 13 k G/cm (1 ms), G_H_ = 13 k G/cm (1 ms). The pulse sequence codes and parameter files for Bruker Avance system are available from authors upon request

Analogously to 4D H^α^-detected experiments described above, the 4D H^N^-detected experiments (Fig. 5), with an isolated proline assignment enhancement, are based on their 3D counterparts (Hellman et al. 2014). Again, sampling of an additional ^15^N dimension can be implemented in without introducing sensitivity loss thanks to the gradient enhanced coherence order selective coherence transfer (COS-CT) (Kay et al. 1992; Schleucher et al. 1994). Magnetization transfer through (HACA)CON(CA)NH and (HACA)N(CA)CONH experiments are briefly described in Eqs. 4 and 5, respectively:

4 $${}^{1}{\text{H}}^{\alpha } (i - 1)\xrightarrow{{2\tau \left( {^{1} J_{{C\alpha H\alpha }} } \right)}}{}^{{13}}C^{\alpha } (i - 1)\xrightarrow{{2T_{C} \left( {^{1} J_{{C\alpha C'}} } \right)}}{}^{{13}}C^{\prime}(i - 1)\left[ {2T_{{C^{\prime}N}} - t_{1} ;{}^{1}J_{{C^{\prime}N}} } \right] \to {}^{{15}}N(i)[t_{2} ] \to {}^{{13}}C^{\alpha } (i - 1)\xrightarrow{{2T_{{CAN}} \left( {{}^{1}J_{{C\alpha N}} ,{}^{2}J_{{C\alpha N}} } \right)}}{}^{{15}}N(i - 1)\left[ {2T_{{NCA}} - t_{3} ;{}^{1}J_{{C\alpha N}} ,{}^{2}J_{{C\alpha N}} } \right]\xrightarrow{{4\Delta \left( {^{1} J_{{NH}} } \right)}}{}^{1}H^{N} (i - 1)[t_{4} ]$$

5 $${}^{1}{\text{H}}^{\alpha } (i)\xrightarrow{{2\tau \left( {^{1} J_{{C\alpha H\alpha }} } \right)}}{}^{{13}}C^{\alpha } (i)\xrightarrow{{2T_{{CN}} \left( {{}^{1}J_{{C\alpha N}} ,{}^{2}J_{{C\alpha N}} } \right)}}{}^{{15}}N(i)[t_{1} ] \to {}^{{13}}C^{\alpha } (i)\xrightarrow{{2T_{{CAN}} ,2T_{C} \left( {{}^{1}J_{{C\alpha N}} ,{}^{2}J_{{C\alpha N}} ,{}^{1}J_{{C\alpha C^{\prime}}} } \right)}}{}^{{13}}C^{\prime}(i)\left[ {2T_{C} - t_{2} ;{}^{1}J_{{C\alpha C^{\prime}}} } \right] \to {}^{{15}}N(i + 1)\left[ {2T_{{NC}} - t_{3} ;{}^{1}J_{{C^{\prime}N}} } \right]\xrightarrow{{4\Delta \left( {^{1} J_{{NH}} } \right)}}{}^{1}H^{N} (i + 1)[t_{4} ]$$

Like in the H^α^-detected experiments (vide supra), the magnetization is first transferred from ^1^H^α^ to ^13^C^α^ spin (time point *a*), and further to either ^13^C′(*i*–1) spin in (HACA)CON(CA)NH (density operator C^α^_z_(*i*–1)C′_y_(*i*–1)) or selectively to ^15^N(*i*) spin in (HACA)N(CA)CONH (density operator C^α^_z_(*i*)N_y_(*i*)) at time point *b*. This is followed by the frequency labeling of ^13^C′(*i*–1) and ^15^N(*i*) chemical shifts during t_1_ in (HACA)CON(CA)NH and (HACA)N(CA)CONH experiments, respectively. Next, the desired magnetization is transferred to the sequential ^15^N spin or intraresidual ^13^C′ spin, described by the density operators C^α^_z_(*i*–1)C′_z_(*i*–1)N_y_(*i*) and C^α^_z_(*i*)C′_y_(*i*)N_z_(*i* + 1) in (HACA)CON(CA)NH and (HACA)N(CA)CONH, respectively (time point *c*). Frequency labeling of ^15^N(*i*) and ^13^C′(*i*) chemical shift takes place in t_2_. The suppression of sequential ^15^N pathway in (HACA)N(CA)CONH is accomplished using the intraresidual filtering between time points *b* and *c*, and in (HACA)CON(CA)NH between time points *c* and *d*. Finally, the chemical shift of ^15^N(*i*–1) and ^15^N(*i* + 1) chemical shifts are recorded for C^α^_z_(*i*–1)N_y_(*i*–1) and C′_y_(*i*)N_z_(*i* + 1) coherences in (HACA)CON(CA)NH and (HACA)N(CA)CONH between time points *d*–*e*.

Hence, the 4D (HACA)CON(CA)NH and (HACA)N(CA)CONH spectra exhibit correlations at the intersection of ω_HN(i–1)_, ω_N(i–1)_, ω_C′(i–1)_, ω_N(i)_ and ω_HN(i+1)_, ω_N(i+1)_, ω_C′(i)_, ω_N(i)_ frequencies, respectively. As the magnetization transfer cascade originates from the H^α^ spin, and the transfer route includes sampling of ^15^N frequencies of prolines as well, the experiments facilitate assignment of isolated proline residues (XP or PX dipeptide stretches, where P is a proline and X stands for any non-proline residue) together with other residues in IDPs with high sensitivity. Analogously to H^α^-detected experiments, under identical conditions (vide supra), the coherence transfer efficiency for both prolines and non-prolines in the 4D (HACA)CON(CA)NH experiment reaches 0.19, if 2T_C_ is set to 28 ms (and 0.15 with 2T_C_ = 6.2 ms) and in the (HACA)N(CA)CONH experiment I ~ 0.20. Hence, both of these experiments are optimal also for the proline assignment in XPX moieties.

**Fig. 6:**
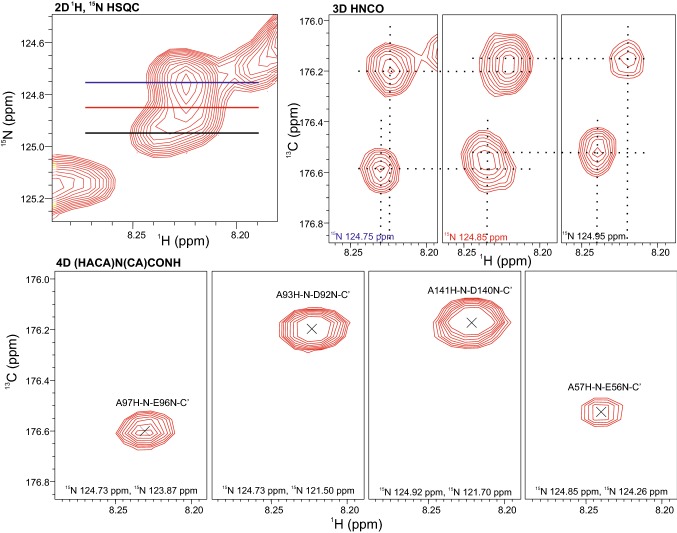
Resolving power of the i-1 amide nitrogen in the fourth dimension. Compared are 3D HNCO and 4D (HACA)N(CA)CONH planes from the ^1^H, ^15^N-HSQC region shown in the upper left corner. From 2D H^N^-C′ planes retrieved from the 3D HNCO at ^15^N frequencies marked with lines of different colors it is difficult to determine the number of peaks present and their chemical shifts in this overlapping peak area of the ^1^H, ^15^N-HSQC. Tentative peak positions are shown with broken lines. In contrast, in the 4D (HACA)N(CA)CONH spectrum four nicely resolved peaks are found. The fourth dimension, i.e. the second chemical shift marked in the 4D planes effectively spreads peaks overlapping in the 2D and 3D spectra

In the ^1^H, ^15^N HSQC only 41% of the residues displayed resolved peaks, whereas from the two 4D spectra, accurate amide ^1^H, ^15^N shifts were obtained for 85% of BilRI residues. Still overlapping peaks were found for residues ^73^Lys-^80^Met, ^113^Lys-^120^Met and ^153^Lys-^154^Ala in the ^71/111/151^EVKDAI/VSEKM^80/120/160^ stretches as well as for residues ^103^Asp-^105^Ala, ^143^Asp-^145^Ala in the middle of the ^99/139^KDAKDAAADKM^109/149^ stretches. Despite the overlap, with the help of the high peak resolution in the CON, it was possible to walk sequentially the whole amino acid sequence with the exception of the first three residues, which are not part of the native BilRI sequence. These residues are missing also from the ^1^H, ^15^N HSQC, likely due to fast chemical exchange of their amide proton with the solvent protons. Examples of the resolving power of the additional ^1^H, ^15^N HSQC dimension are presented in Fig. 6 and Suppl. Figs. S3, S4. The sequential walk over a proline residue is depicted in Suppl. Fig. S5.

It is interesting to note that throughout the identical stretches ^71/111^EVKDAIVSEKMDAM^83/123^ and ^99/139^KDAKDAAADKM^109/149^ the corresponding peaks are resolved in the CON spectrum. Naturally, the chemical shift differences become small in the middle of the repeats, ~ 0.03–0.04 ppm for both C′ and N^H^, but the peaks remain, however, distinct. ^76^/^116^Ile C^α^-H^α^ is resolved into separate peaks in a high-resolution CT ^1^H, ^13^C-HSQC spectrum (t_1_ acquisition time 51 ms) as well. To have a different chemical shift, the corresponding atoms in the stretches should experience a mutually different structural environment. Future studies will uncover whether this arises from nearest-neighbor effects being effective over five residues or from distinct long-range interactions within the protein.

During the past several years, several different NMR experiments and assignment schemes have been developed for studying IDPs. Based on the detected spin, these can be categorized to three different classes i.e. H^N^-, ^13^C′- and H^α^-detection approaches (for review, see e.g., Brutscher et al. 2015). The H^N^-detected experiments offer the highest sensitivity under acidic conditions and can be combined with the BEST and TROSY implementations (Solyom et al. 2013; Brutscher et al. 2015). However, they show limited performance under alkali conditions and/or with IDPs having high proline content, which can be partially compensated using an H^α^-start, H^N^-detected approach as shown here and also previously demonstrated (Mäntylahti et al. 2009; Hellman et al. 2014; Yoshimura et al. 2015). For instance, Yoshimura et al. (2015) proposed ^13^C′-^13^C′ TOCSY transfer in HNCOCONH -type experiments which show connectivities for *i*, *i* ± 1, *i* ± 2 residues i.e. enabling connection of *i* and *i* ± 2 residues over a single proline at *i* ± 1 position. The (HACA)CON(CA)NH and (HACA)N(CA)CONH experiments proposed here link the residues flanking a single proline through its ^15^N chemical shift.

Unlike H^N^-detected experiments, the ^13^C-detection is almost non-susceptible to amide proton exchange with water at high pH. The ^13^C-detection based experiments for the assignment of IDPs generally apply the ‘CON′ strategy for connecting the neighboring residues in low-complexity regions of IDPs, which is also suitable for the assignment of prolines (Pantoja-Uceda and Santoro 2014; Sahu et al. 2014; Brutscher et al. 2015; Chaves-Arquero et al. 2018). Theoretical coherence transfer efficiency in the ^13^C-detected experiment, hacaCONcaNCO (Pantoja-Uceda et al. 2014) attains 0.19, which is comparable to the sensitivity of the (HACA)CON(CA)NH experiment proposed here. However, given that γ_H_/ γ_C_ equals 4 and the attainable sensitivity has γ^3/2^ dependence on the detected spin, the ^13^C-detection results in an inherent sensitivity loss by a factor of 8 with respect to the ^1^H-detection. This can partially be redeemed using a ^13^C-detection optimized probehead and ^13^C′-detection, which offers higher resolution in the direct detection dimension due to absence of homonuclear couplings. If such a probehead with an inner coil for ^13^C is not available, this sets limit to the feasible sample concentration (> 0.3–0.5 mM) which might become an issue with some IDPs. On the other hand, homonuclear couling between H^N^ and H^α^ can be removed in ^1^H-detected experiments using the homonuclear BASH decoupling scheme (Ying et al. 2014).

Finally, the H^α^-detected experiments offer higher sensitivity with respect to the ^13^C-detection, enable assignment of consecutive prolines and are not prone to linebroadening due to chemical exchange with the solvent (Mäntylahti et al. 2010, 2011). However, given that H^α^ resonances may partially be underneath the water signal unless sample is prepared in 99.99% D_2_O, all experiments proposed here employ the sensitivity-enhanced, echo/antiecho gradient selection in conjunction with the water flip back approach. This enabled us to carry out all the measurements in 95% H_2_O and accomplish a nearly complete assignment of H^α^ resonances of the 165-residue BilRI. When working with more dilute samples (< 0.25 mM), dissolving protein into D_2_O might be advantageous.

In all, with the proposed suite of 4D experiments, a complete assignment of backbone H^N^, N^H^, C′ chemical shifts was obtained for all but the three N-terminal residues, which are not part of the BilRI native sequence. H^α^, C^α^ and C^β^ chemical shifts were obtained for 87, 76, and 66% of the residues, respectively. These experiments offer alternative approach for the assignment of IDPs. Hence, they should not be considered superior per se to the existing methods, but as demonstrated here, they have features that may facilitate accomplishing the assignment of challenging IDPs such as BilRI.

## Conclusions

In this paper, we have presented a suite of H^α^-detected and H^α^-start, H^N^-detected 4D experiments that by exploiting chemical shift dispersion brought by an additional C^α^ or N dimension, facilitate the unambiguous backbone resonance assignment of IDPs in comparison to their established 3D counterparts. The H^α^-detected experiments provide versatile strategy for the assignment of proline-rich amino acid sequences and/or proteins at alkali pH, prone to fast chemical exchange with the solvent. The proposed 4D H^α^-detected experiments provide information with the most vital chemical shifts i.e. H^α^, ^13^C^α^, and ^13^C′ for conformational restraining in IDPs. The H^α^-start, H^N^-detected (HACA)CON(CA)NH and (HACA)N(CA)CONH experiments offer an alternative assignment protocol for IDPs, by providing connectivities over three sequential residues with the H^N^-detection that allows for connecting single proline-containing segments of the amino acid sequence. The feasibility of the proposed experiments was experimentally verified on with BilRI, a 165-residue protein, containing three highly similar 40-residue repeats and 12 K-D-A triplet sequences, which represents an extremely challenging case of resonance assignment of IDPs.

## Electronic supplementary material

Below is the link to the electronic supplementary material.

Supplementary file1 (PDF 2150 kb)
