# Supplementary material for: Dispersion from Cα or NH: 4D experiments for backbone resonance assignment of intrinsically disordered proteins
Source: J Biomol NMR. 2020 Jan 13;74(2):147–59. doi: 10.1007/s10858-020-00299-w (PMC7080685; doi:10.1007/s10858-020-00299-w)
Supplement: Supplementary file 1 — Supplementary file1 (PDF 2150 kb) [file 10858_2020_299_MOESM1_ESM.pdf]

Supplementary Material

Dispersion from C^{α} or N^{H}: 4D experiments for backbone resonance assignment of intrinsically disordered proteins

Helena Tossavainen[1], Santeri Salovaara[1], Maarit Hellman[1], Riikka Ihalin[2], Perttu Permi[1,3*]

[1]Department of Chemistry, Nanoscience Center, University of Jyväskylä, FI-40014 Jyväskylä, Finland.
[2]Department of Biochemistry, University of Turku, FI-20014 Turku, Finland.
[3]Department of Biological and Environmental Science, University of Jyväskylä, FI-40014 Jyväskylä, Finland

*Corresponding author:
Perttu Permi, perttu.permi@jyu.fi

ORCID of the authors:
Helena Tossavainen, 0000-0002-1609-1651
Riikka Ihalin, 0000-0003-4118-0370
Perttu Permi, 0000-0002-6281-1138

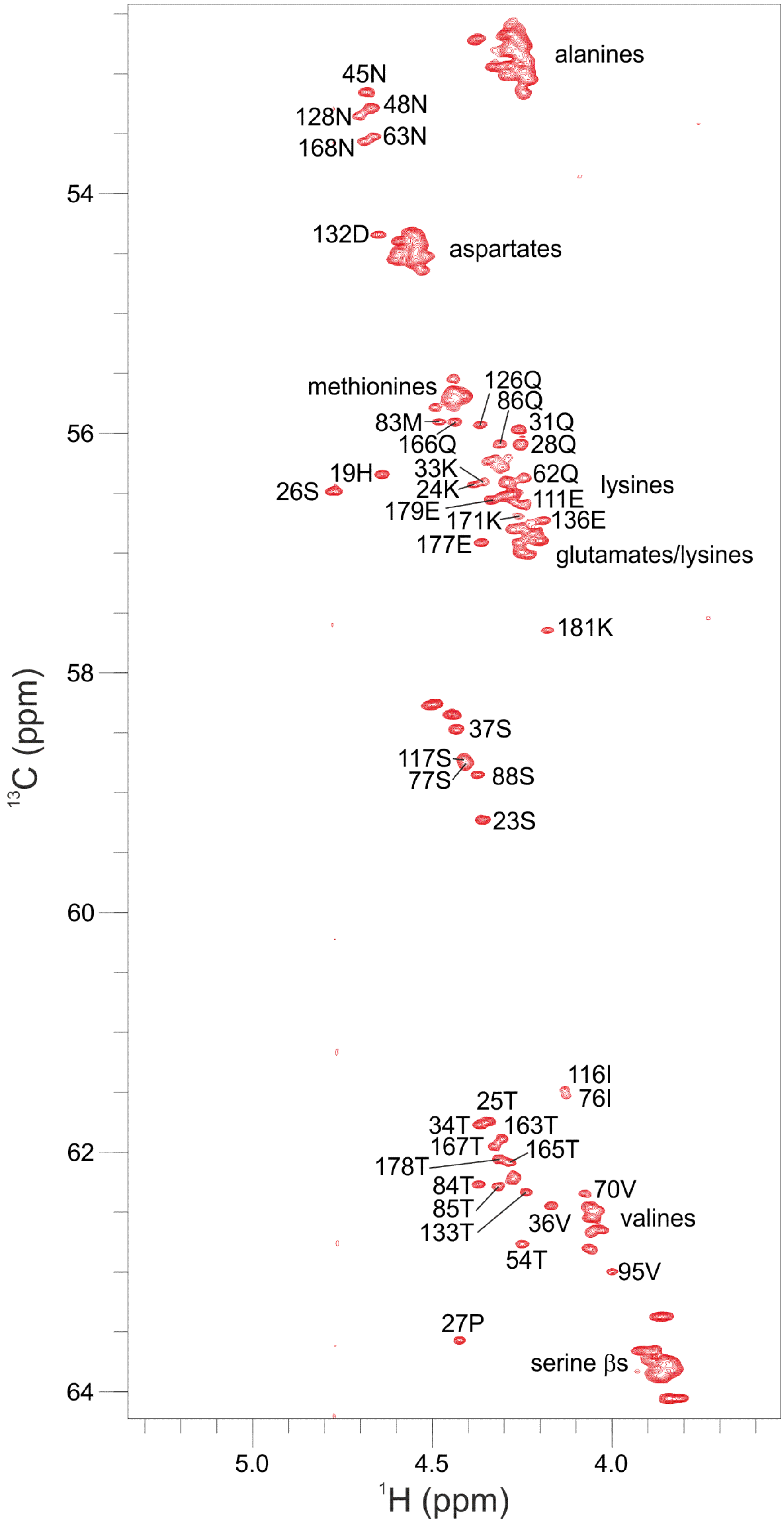

Suppl. Fig. S1 The C^{α}/H^{α} region of the ^{1}H, ^{13}C CT HSQC of BilRI measured at 800 MHz ^{1}H frequency, 25 °C, 1 mM, pH 6.5. The resolved signals are labeled with sequence number and amino acid code.

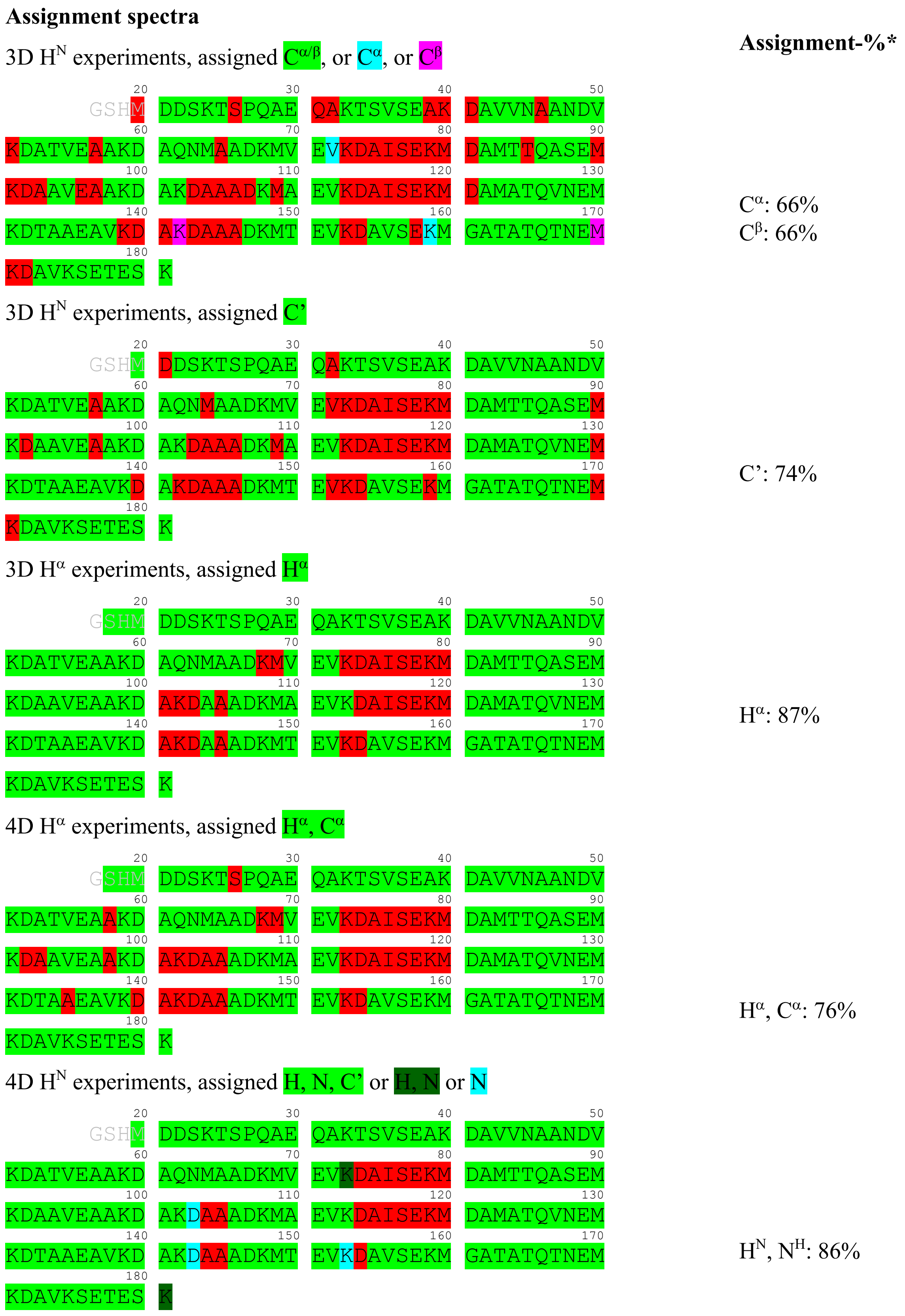

Suppl. Fig. S2. Assignment coverages and percentages from spectra used in the BilRI assignment. *The N-terminal cloning artefact GSHM is excluded.

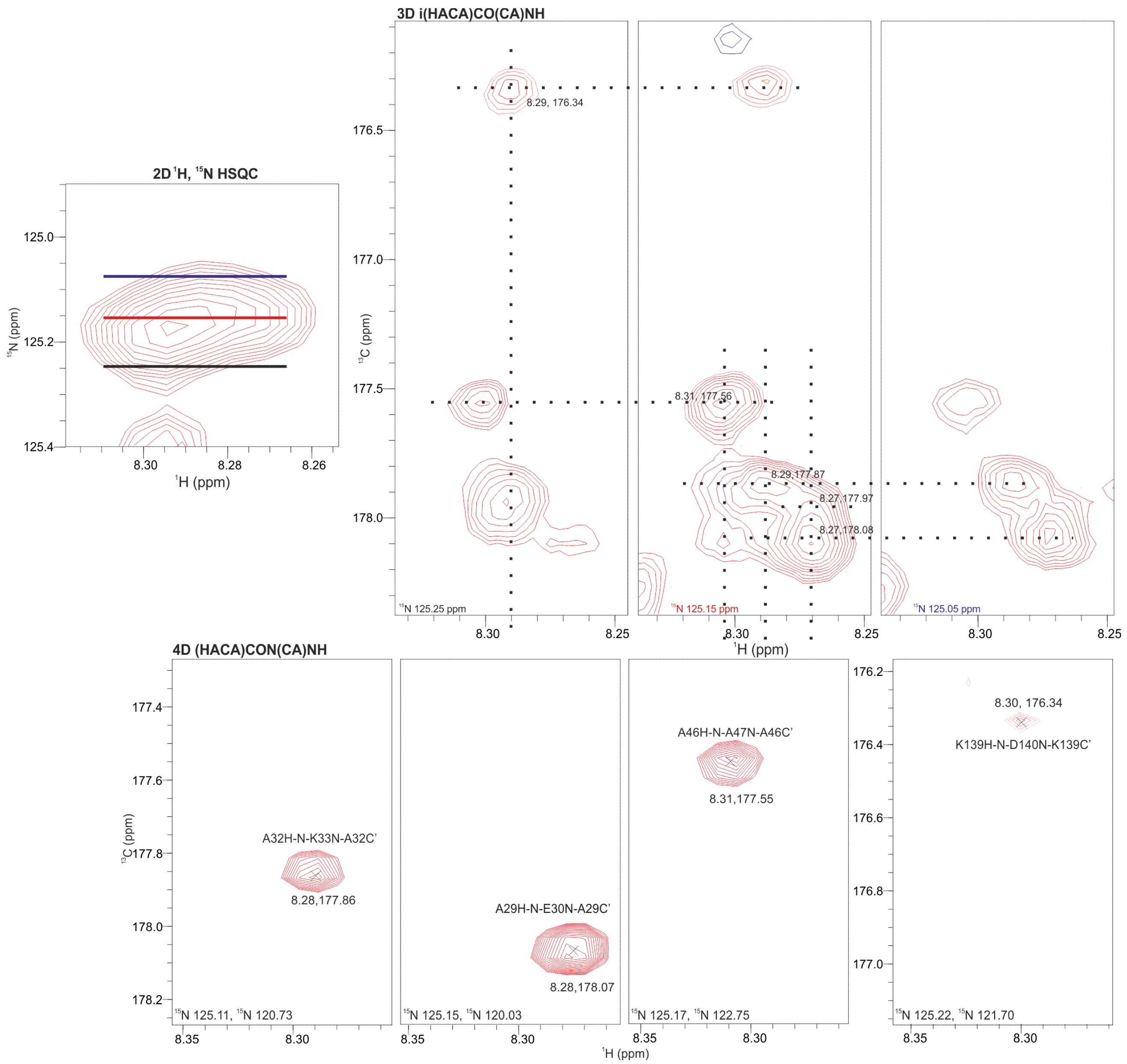

Suppl. Fig. S3 Similar to Fig. 1 of the main text, the **r**esolving power of the i+1 amide nitrogen in the fourth dimension is presented. Compared are 3D i(HACA)CO(CA)NH and 4D (HACA)CON(CA)NH planes from the ^{1}H, ^{15}N-HSQC region at upper left corner. From 2D H^{N}-C' planes of the 3D i(HACA)CO(CA)NH at ^{15}N frequencies marked with lines of different colors it is difficult to determine the number and chemical shifts of peaks present in the overlapping peak area in the ^{1}H, ^{15}N-HSQC. Tentative shifts are marked for five peaks. In contrast, in the 4D (HACA)CON(CA)NH spectrum four nicely resolved peaks are found.

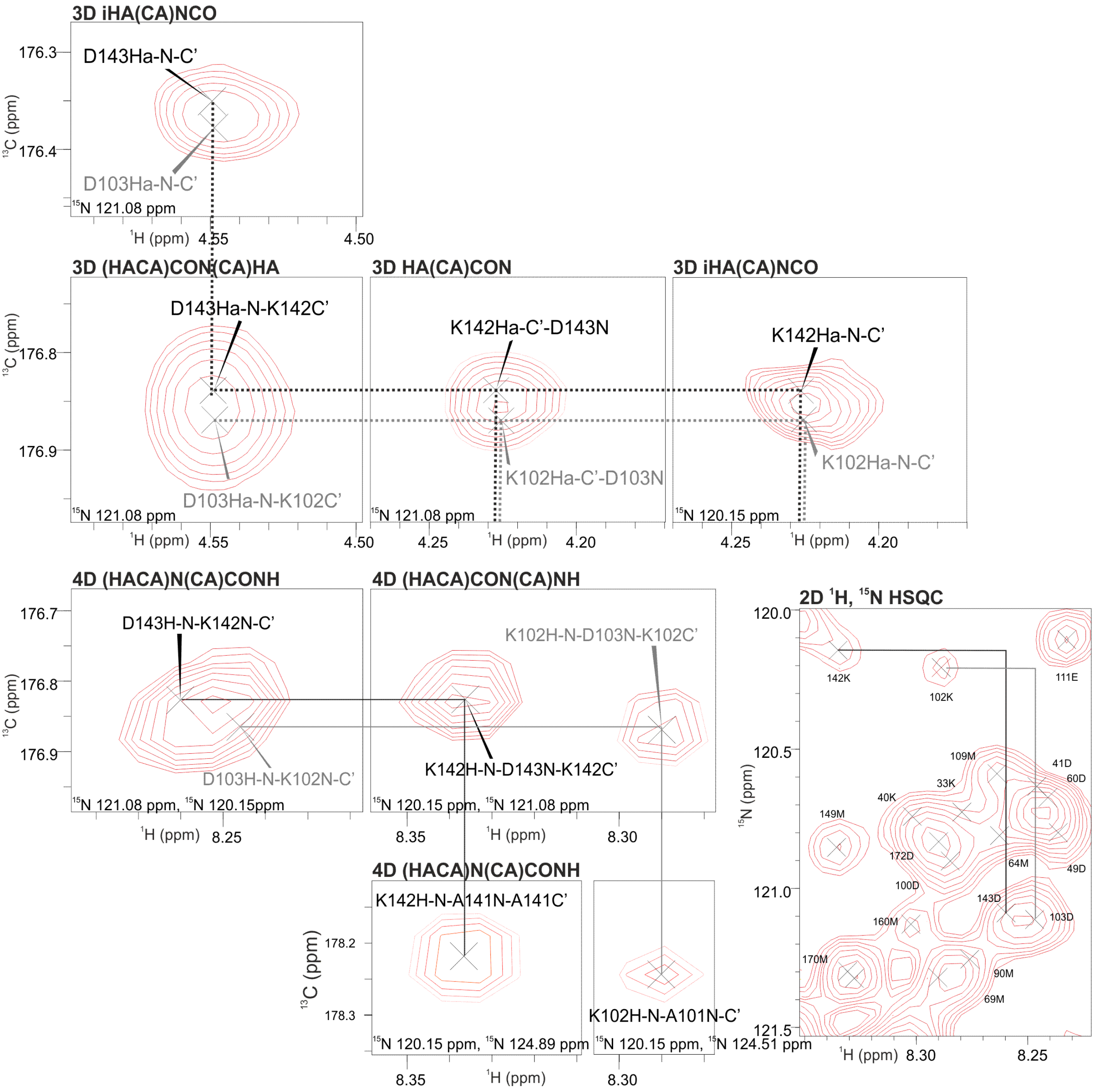

Suppl. Fig. S4 Representative planes from 4D (HACA)N(CA)CONH and (HACA)CON(CA)NH spectra, which demonstrate the resolving power of sequential N^H in resolving ambiguities present in 3D H^{α}-detected experiments. Shown is the assignment of ^{102}Lys-^{103}Asp/^{142}Lys-^{143}Asp in the KDA**KD**AAADKM stretches (residues 99-106 and 139-146). The intraresidual and sequential H^{α}, N^H, C' correlations of ^{103}Asp and ^{143}Asp appear as one peak in the three H^{α}-detected experiments, and their assignments remain ambiguous. Although the ^{143}AspH,N-^{142}Lys N,C/^{103}AspH,N-^{102}LysN,C' correlations are not resolved in the 4D (HACA)N(CA)CONH, thanks to distinct H^N shifts, the preceding residues display well separated peaks in the 4D (HACA)CON(CA)NH, which allows to proceed with the sequential walk. The sequential walk is presented in the ^{1}H, ^{15}N HSQC spectrum at the lower right corner. Grey labels mark correlations with peak maximum in adjacent ^{15}N/^{15}N, ^{13}C planes.

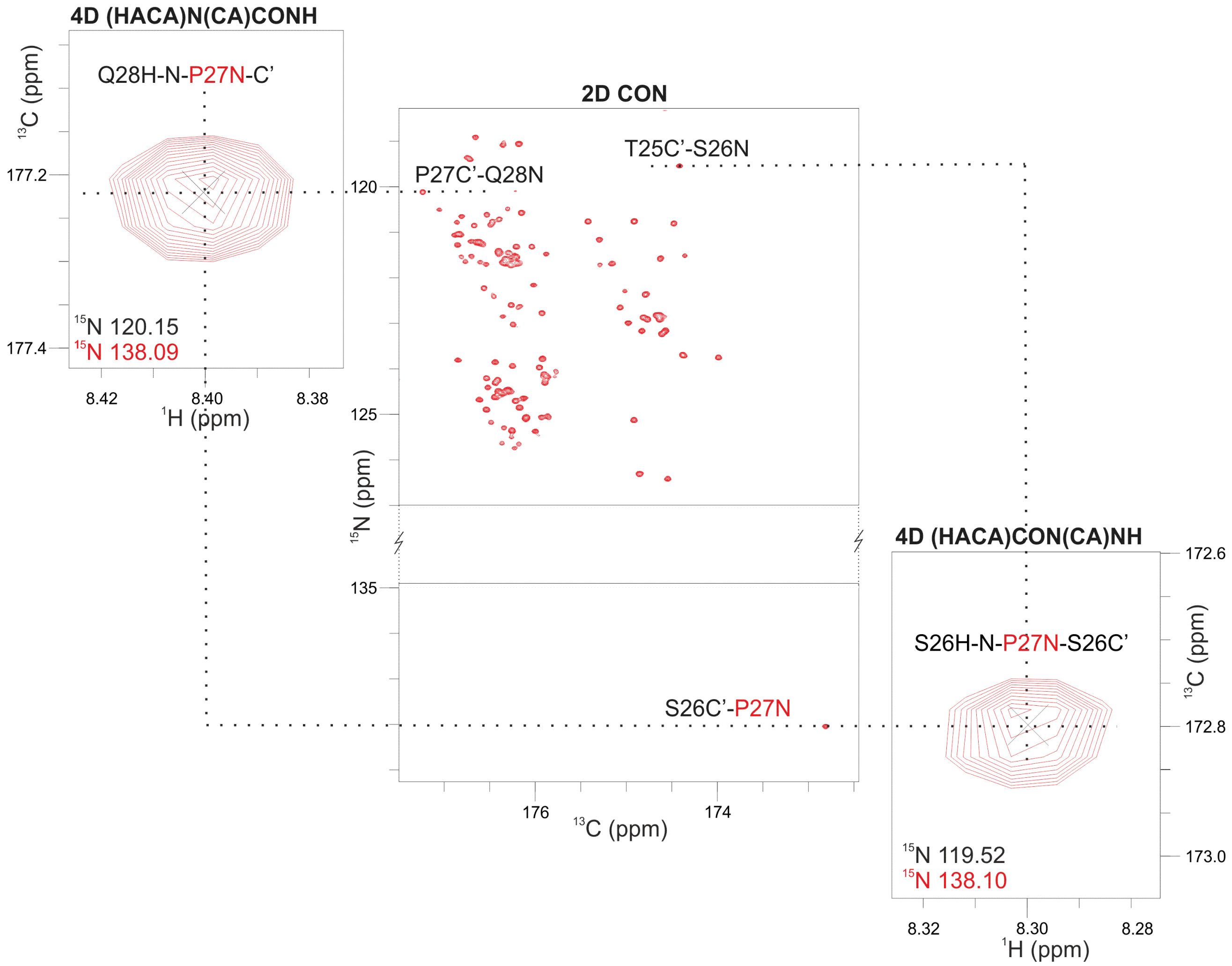

Suppl. Fig. S5 Assignment of proline-containing amino acid sequences using 4D (HACAN(CA)CONH and (HACA)CON(CA)NH. Shown is the assignment of ^{28}Gln-^{27}Pro-^{26}Ser triplet in BilRI. The (HACA)N(CA)CONH shows a correlation between ^{28}Gln H^N, N^H and N^H, C' of the preceding proline. At this proline N^H chemical shift in the (HACA)CON(CA)NH the H^N, N^H, C' of the preceding ^{26}Ser. The corresponding peaks and the pathway between them are shown in the 2D CON spectrum.

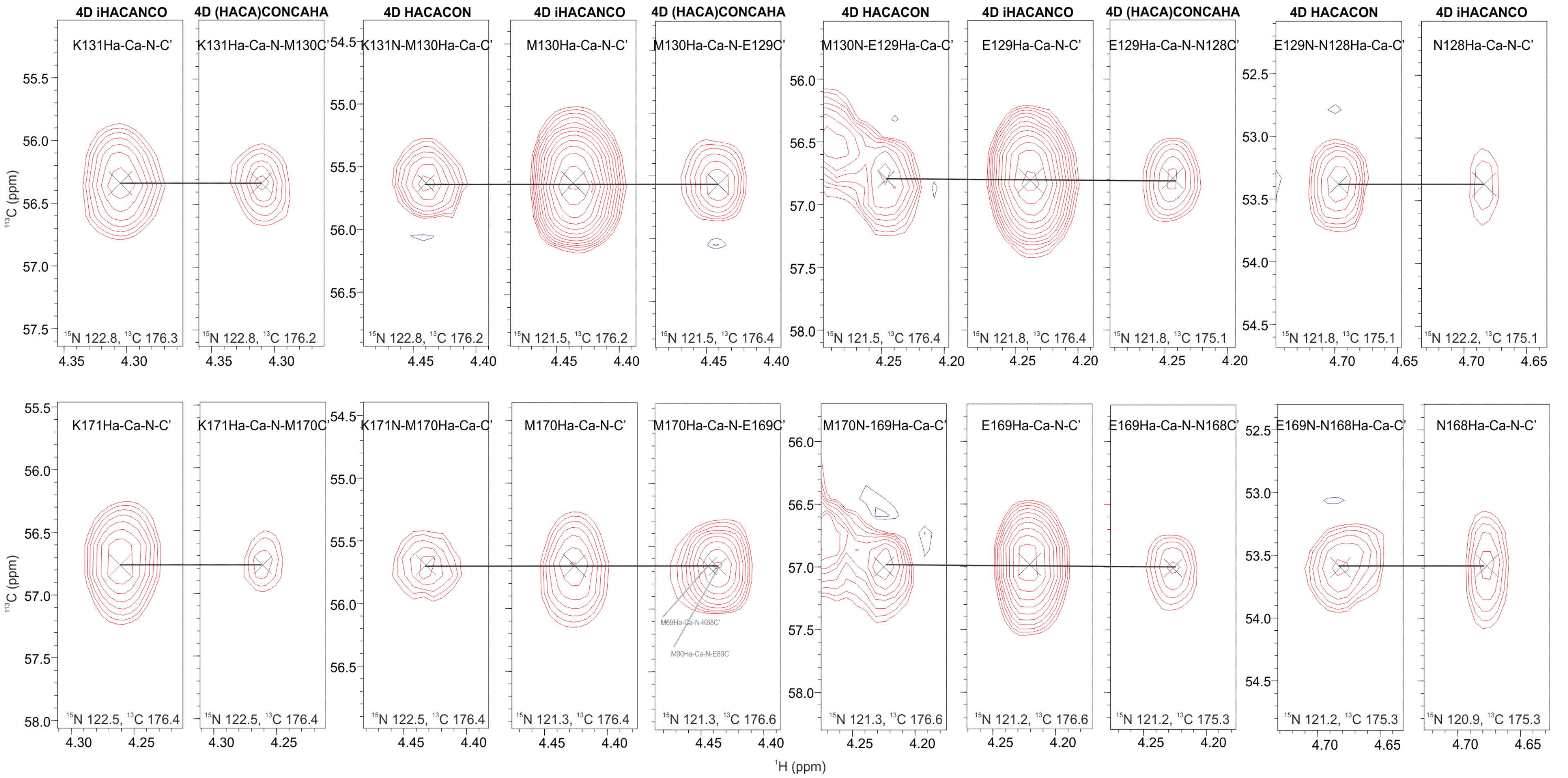

Suppl. Fig. S5 Assignment of identical stretches ^{128}Asn-^{129}Glu-^{130}Met-^{131}Lys and ^{168}Asn-^{169}Glu-^{170}Met-^{171}Lys using the described three 4D experiments iHACANCO, (HACA)CONCAHA and HACACON.
